# Stability of Phytochemical Compounds and Antioxidant Capacity of Garambullo (*Myrtillocactus geometrizans*) Extract Microencapsulated with Different Concentrations of Wall Material (Gum Arabic) During Storage and In Vitro Digestion

**DOI:** 10.3390/foods15162900

**Published:** 2026-08-19

**Authors:** Joselyn Estefanía Quiroz Zarco, Ofelia Gabriela Meza-Márquez, Guillermo Osorio-Revilla, Tzayhri Gallardo-Velázquez, Oswaldo Arturo Ramos-Monroy

**Affiliations:** 1 Instituto Politécnico Nacional, Escuela Nacional de Ciencias Biológicas-Zacatenco, Departamento de Ingeniería Bioquímica, Wilfrido Massieu 399, Nueva Industrial Vallejo, Zacatenco, Alcaldía Gustavo A. Madero, Ciudad de México C.P. 07738, Mexico; jquirozz1400@alumno.ipn.mx (J.E.Q.Z.); oramosm@ipn.mx (O.A.R.-M.); 2 Instituto Politécnico Nacional, Escuela Nacional de Ciencias Biológicas-Santo Tomás, Departamento de Biofísica, Prolongación de Carpio y Plan de Ayala s/n. Santo Tomás, Alcaldía Miguel Hidalgo, Ciudad de México C.P. 11340, Mexico; tgallardov@ipn.mx

**Keywords:** phytochemical compounds, garambullo, *Myrtillocactus geometrizans*, microencapsulation, spray drying, gum arabic, storage, in vitro simulated digestion

## Abstract

The garambullo fruit (*Myrtillocactus geometrizans*) is rich in phytochemical compounds; however, once these phytochemical compounds are extracted from the fruit, they are susceptible to degradation caused by environmental conditions, storage and the digestive process. The objective of the present study was to microencapsulate the ethanolic extract of garambullo fruit through spray drying using gum arabic (GA) at concentrations of 10%, 15% and 20%, and to determine its effect on the stability of phenolic compounds (PC), betacyanins (Bc), betaxanthins (Bx) and antioxidant capacity (ABTS; FRAP assays), during both storage and in vitro digestion. Storage at three temperatures (4 °C, 18 °C, and 30 °C) revealed that the degradation of phytochemical compounds and antioxidant capacity depends on both storage temperature and the concentration of the wall material. Through chemical kinetic analysis, it was determined that the degradation of phytochemical compounds and antioxidant capacity during storage followed first-order reaction kinetics. Microcapsules with 20% GA stored at 4 °C exhibited the longest half-life of phytochemical compounds and antioxidant capacity (PC: 3465 days; Bc: 1179 days; Bx: 1732 days; ABTS: 2590 days; and FRAP: 1821 days). After in vitro digestion, microcapsules with 20% GA exhibited the highest bioaccessibility of phytochemical compounds and antioxidant capacity (PC: 94.91%, Bc: 31.51%, Bx: 58.50%; ABTS: 89.17%, and FRAP: 88.46%), followed by microcapsules formulated with 15% GA (PC: 83.71%, Bc: 26.86%, Bx: 49.89%, ABTS: 74.31%, and FRAP: 80.64%), and finally microcapsules formulated with 10% GA (PC: 66.11%, Bc: 19.97%, Bx: 37.08%, ABTS: 63.45%, and FRAP: 52.57%). These results are intended to promote the use of garambullo fruit in the food industry for the formulation of functional foods or nutraceuticals, thereby enabling the exploitation of the benefits associated with the antioxidant compounds present in this fruit.

## 1. Introduction

Garambullo (*Myrtillocactus geometrizans*) is an endemic cactus species of Mexico that is widely distributed throughout the country. However, its utilization remains locally restricted because it is a highly perishable fruit, resulting in losses of up to 70% of its production. Nevertheless, the fruit is a good source of phytochemical compounds, including betalains, which have been extensively studied because of their antioxidant potential [1].

Phytochemical compounds present in garambullo fruit, once extracted, are susceptible to degradation due to various factors such as light, temperature, oxygen exposure and gastrointestinal process [2]. For this reason, for their potential application, it is imperative to protect these compounds using techniques such as microencapsulation.

Microencapsulation by spray drying is commonly used for the preservation of phytochemical compounds, since during the process the active ingredients are protected by a wall material [3]. The choice of wall material is an important factor which is based on physicochemical properties (solubility, molecular weight and emulsifying properties) and must be generally regarded as safe and biodegradable, and should not affect the taste or flavor of the encapsulated material. The wall materials commonly used in spray drying include proteins (soybeans, barley, casein, and gelatin) and polysaccharides (maltodextrin, gum arabic, guar gum, cellulose, pectins, carrageenan, alginates, and chitosan) [4,5,6].

Among the above, gums stand out because they offer the advantage of being abundant in nature and low in cost. In addition, this type of material provides biocompatibility and biological activity to the food matrix. One of the most widely used gums in microencapsulation is gum arabic (GA) because it is odorless, tasteless, does not interact with other compounds, exhibits good solubility, low viscosity, low water absorption capacity and low cost [5]. In addition, gum arabic is a natural polysaccharide that is not digestible and acts as insoluble fiber, unlike other wall materials such as maltodextrin, which is a polysaccharide derived from starch and contains a high amount of glucose, which must be taken into account if this microencapsulated extract is used for patients with diabetes mellitus.

Several authors have studied the effect of gum arabic on the stability of phytochemical compounds in various food matrices [7,8,9]. Ruiz-Aguilar et al. [8] microencapsulated garambullo extract using 10% gum arabic in the feed solution, and it was found that during storage at 30 °C, the microcapsules lost approximately 60% of the phytochemical compounds. Likewise, at the end of in vitro digestion, the bioaccessibilities of phenolic compounds, betalains and antioxidant capacity (ABTS) were 65%, 25% and 40% respectively.

Based on the above, the present study aimed to improve the results reported by Ruiz-Aguilar et al. [8] by increasing the concentration of the wall material (GA) to 15% and 20% in the feed solution and evaluating its effect on the degradation of phytochemical compounds and antioxidant capacity, through chemical kinetics at 4 °C, 18 °C and 30 °C over a period of 60 days. Likewise, the effect of wall-material concentration on the bioaccessibility of phytochemical compounds and antioxidant capacity was analyzed by in vitro digestion.

This study sought to obtain the most appropriate wall-material (GA) concentration to ensure maximum protection of the microencapsulated phytochemical compounds present in garambullo extract during storage, as well as to increase the bioaccessibility of compounds after in vitro digestion.

## 2. Materials and Methods

### 2.1. Reagents

All reagents were of analytical grade. Reagents included 2,4,6-Tris(2-pyridyl)-s-triazine (TPTZ) (Sigma-Aldrich Co. LLC., St. Louis, MO, USA), 2,2′-azino-bis(3-ethylbenzothiazoline-6-sulfonic acid) (ABTS) (Sigma-Aldrich Co. LLC.), sodium bicarbonate (Realyt’s, Mexico City, Mexico), 0.3 M acetate buffer (pH 3.6), sodium carbonate (Realyt’s), gallic acid (Sigma-Aldrich Co. LLC.), ethanol (Reasol, Mexico City, Mexico), hexane (Reasol), potassium persulfate (Fermont, Mexico City, Mexico), Folin–Ciocalteu reagent (Sigma-Aldrich Co. LLC.), anhydrous sodium sulfate (Reasol), trolox (Sigma-Aldrich Co. LLC.), α-amylase from porcine pancreas (Catalog number A3176, CAS number 9000-90-2, EC number 3.2.1.1, Sigma-Aldrich Co. LLC.), bile extract porcine (Catalog number B8631, CAS number 8008-63-7, EC 232.369.0, Sigma-Aldrich Co. LLC.), pancreatin from porcine pancreas (Catalog number P1750, CAS number 8049-47-6, EC 232.468.9, Sigma-Aldrich Co. LLC.), pepsin from porcine gastric mucose (Catalog number P7000, CAS number 9001-75-6, EC 3.4.23.1, Sigma-Aldrich Co. LLC.), and gum arabic (Agricurama S.P.R. de R.L. de C.V., Mexico City, Mexico).

### 2.2. Acquisition, Conditioning, and Processing of Garambullo Fruits

Garambullo fruits (*M. geometrizans*) were acquired in the municipality of Santiago Valley (Guanajuato, México) at the mature stage (purple coloration). The fruits were washed with running water and dried on metal trays in an oven (Blue Lindberg, V0914A, Thermo Scientific, Waltham, MA, USA) set at 60 °C for 24 h. Subsequently, the dried fruits were ground using a food processor (Osterizer^®^ MAX, BLSTPBRGR, Los Angeles, CA, USA), sieved through a #50 mesh and stored protected from light until use.

### 2.3. Extraction and Concentration of Antioxidant Compounds

Extraction of phytochemical compounds was performed according to the methodology described by Ruiz-Aguilar et al. [8]: powdered garambullo was mixed with 30% ethanol:water (*v*/*v*) at a 1:10 ratio (p/*v*). Extraction was carried out at 30 °C for 30 min. The mixture was then centrifuged (3500 rpm) and the supernatant was separated. This procedure was repeated once more to obtain two successive extractions. The supernatant obtained from the second extraction was combined with that from the first extraction and both were filtered through Whatman #4 filter paper. Finally, the filtered extract was concentrated using a rotary evaporator (Büchi, I-300 Pro, Flawil, Switzerland) to remove the solvent (ethanol). The concentrated extract was analyzed for phenolic compounds, betacyanins, betaxanthins, and antioxidant capacity, and was subsequently used for microencapsulation.

### 2.4. Analytical Techniques

#### 2.4.1. Phenolic Compounds (PC)

Phenolic compounds (PC) were determined using the methodology described by Singleton and Rosi [10]. Briefly, 0.1 mL of sample was mixed with 3.9 mL of distilled water and 0.25 mL of 50% Folin–Ciocalteu reagent, then vortexed (Cole-Palmer, Scout SC2020, Beijing, China) and allowed to stand for 6 min. Subsequently, 0.75 mL of 20% sodium carbonate was added and vortexed. The samples were then stored at room temperature in the dark for 1 h. Finally, absorbance was measured using a spectrophotometer (Jenway, 6320D, Staffordshire, UK) at 750 nm. The blank consisted of the reagent mixture without the sample. The calibration curve (0.1–0.5 mg/mL, n = 5) was constructed using gallic acid as the standard. Results were expressed as milligrams of gallic acid equivalents (GAE) per 100 g of dry solids (mg GAE/100 g_dw_).

#### 2.4.2. Betacyanins (Bc) and Betaxanthin (Bx)

Bc and Bx were determined according to the method proposed by MoBhammer et al. [11]. Briefly, 0.1 mL of sample was diluted to a final volume of 3 mL with distilled water, and the absorbance (538 nm for Bc and 480 nm for Bx) was measured using a spectrophotometer (Jenway, 6320D). Bc and Bx content was determined using Equation (1):

(1) $$Bc\;or\;Bx\;\left(mg/g\right)=\frac{A\times DF\times MW\times 1000}{\varepsilon \times l}$$

where:

A: Absorbance of betacyanins (538 nm) or betaxanthins (480 nm).
DF: Dilution factor.
l: Path length of the cuvette (1 cm).
MW: Molecular weight of betanin (550 g/mol) and indicaxanthin (308 g/mol).
ε: Molar extinction coefficient of betanin (60,000 L/mol*cm) or indicaxanthin (48,000 L/mol*cm).

#### 2.4.3. Antioxidant Capacity (AOX)

AOX was determined using two methods: ABTS (2,2′-azino-bis(3-ethylbenzothiazoline-6-sulfonic acid)) as described by Brand-Williams et al. [12] and FRAP (Ferric Reducing Antioxidant Power), as proposed by Benzie and Strain [13]. For both methods, the results were expressed as µmol Trolox equivalents (TE) per 100 g of dry weight (µmol TE/100 g_dw_).

The ABTS assay consisted of generating the ABTS radical (7 mM) and adjusting its absorbance to 7 ± 0.01 at 734 nm using ethanol. Ethanol was used as the blank. A 30 μL aliquot of the sample was mixed with 3 mL of the ABTS radical solution. The test tubes were vortexed and allowed to stand for 6 min. Absorbance was measured at 734 nm. The calculated percentage of inhibition was interpolated using the Trolox standard curve (0.2–2.0 µmol TE/mL, n = 10).

The FRAP assay consisted of preparing the FRAP reagent. A 90 μL aliquot of the sample was taken, followed by the addition of 270 μL of distilled water and 2.7 mL of FRAP reagent. The mixture was vortexed and incubated at 37 °C for 25 min. Absorbance was measured at 593 nm. The blank consisted of the reagent mixture without the sample. Absorbance values were interpolated using the Trolox standard curve (0.1–2.0 µmol TE/mL, n = 7).

### 2.5. Microencapsulation

The concentrated extract was microencapsulated using a spray dryer (GEA Basic Mobile Minor 2000, GEA Group, GEA-NIRO Atomizer, Mobile Minor, Copenhagen, Denmark) at an inlet temperature (Ti) of 170 °C and an outlet temperature (To) of 80 °C [8].

A 30% gum arabic (GA) solution was prepared. The solution was to stand for 24 h at room temperature to eliminate bubbles generated during its preparation. Using this solution, mass balances were performed to determine the proportions of GA and concentrated extract required to obtain 10%, 15% and 20% GA in the solution to be dried. Thereby, the proportions of concentrated extract (CE) and gum arabic (GA) required to obtain concentrations of 10%, 15% and 20% in the solutions to be dried were as follows: 10% (CE 66.66 mL:33.33 mL GA), 15% (CE 50 mL:50 mL GA), and 20% (CE 33.33 mL:66.66 mL GA).

The yield after microencapsulation was calculated using Equation (2).

(2) $$Yield\;(\%)=\frac{Amount\;of\;solids\;recovered}{Amount\;of\;expected\;solids}\times 100$$

The amount of expected solids is obtained from the mass balance performed before drying.

The microcapsules prepared with 10%, 15% and 20% GA were characterized by analyzing the contents of PC, Bc, Bx and AOX (ABTS; FRAP). Retention efficiency (%RE) of phytochemical compounds and AOX was calculated using Equation (3).

(3) $$\%RE=\left(\frac{Content\;of\;phytochemical\;compounds\;or\;AOX\;of\;the\;microcapsules/g_{dw}}{Content\;of\;phytochemical\;compounds\;or\;AOX\;of\;the\;solution\;to\;be\;dried/g_{dw}}\right)\times 100$$

Surface phytochemical compounds of microcapsules were determined using the method proposed by Robert et al. [14]. Briefly, 0.4 g of microcapsules were washed with 4 mL of ethanol:methanol (1:1) and stirred for 1 min. Subsequently, the mixture was centrifuged at 3500 rpm for 10 min. Finally, the content of phytochemical compounds was determined in the supernatant.

Encapsulation efficiency (%EE) of phytochemical compounds and AOX was calculated using Equation (4).

(4) $$\%EE=\left(\frac{Phytochemical\;compounds\;or\;AOX\;in\;microcapsules/g_{dw}-Surface\;phytochemical\;compounds\;or\;AOX/g_{dw}}{Phytochemical\;compound\;content\;or\;AOX\;in\;the\;solution\;to\;be\;dried/g_{dw}}\right)\times 100$$

Moisture content was determined using a moisture analyzer (Ohaus, MB200, Montville, NJ, USA) at 110 °C programmed with a moisture variation of 0.001 g/min for 90 s. Water activity (aw) was determined using an AquaLab (Cx-2, Decagon, Pullman, WA, USA).

Particle size and particle size distribution were determined using a Malvern laser diffraction particle size analyzer (IM 026 serie 2006, Malvern Panalytical, Malvern, UK) equipped with a 100 mm lens. Hexane was used as the dispersing fluid, and acetone was used as the cleaning fluid.

Morphology of the microcapsules was determined using a high-vacuum scanning electron microscope (FEI Quantia FEG250, Thermo Fisher Scientific, Peabody, MA, USA) at 5.0 kV. Samples were uniformly distributed with a spatula onto a conductive tape and coated with gold. Magnifications of 1000× and 2000× were used.

### 2.6. Phytochemical Compounds During Storage

Microcapsules obtained with 10%, 15% and 20% GA were stored in amber bottles at 4 °C, 18 °C and 30 °C (Ecoshel, 9025H, Waltham, MA, USA) for 60 days. Content of PC, Bc, Bx and AOX (ABTS; FRAP) was analyzed every 15 days. Rate constants (*k*) and half-life (t ½) for reaction orders 0, 1, and 2 for the degradation of phytochemical compounds and antioxidant capacity of microcapsules with GA at 10%, 15%, and 20% stored at 4 °C, 18 °C, and 30 °C were determined using Equations (5)–(7).
**Orden****Rate Constants (*****k*****)****Half-Life (t ½)****Linearized Equation**
0$\frac{d{\left[A\right]}_{0}}{dt}=-k$$t\;½=\frac{{\left[A\right]}_{0}}{2k}$[A]_t_ = [A]_0_ − ktEquation (5)1$\frac{d{\left[A\right]}_{0}}{dt}=-k\left[A\right]$$t\;½=\frac{ln\;2}{k}=\frac{0.693}{k}$Ln[A]_t_ = Ln[A]_0_ − ktEquation (6)2$\frac{d{\left[A\right]}_{0}}{dt}=-k{\left[A\right]}^{2}$$t\;½=\frac{{\left[A\right]}_{0}}{2k}$$\frac{1}{{\left[A\right]}_{t}}=\frac{1}{{\left[A\right]}_{0}}-kt$Equation (7)*d*[*A*]_0_: derivative of the initial concentration; dt: derivative of time; k: rate constant; and t ½: half-life.

### 2.7. In Vitro Digestion Process

The bioaccessibility of phytochemical compounds and antioxidant capacity was determined according to the methodology by Minekus et al. [15]. The in vitro digestion process was carried out using the concentrated extract (as a reference) and the microcapsules obtained with 10%, 15% and 20% GA.

For the oral phase, 5 g of sample, 3.5 mL of simulated salivary fluid, 0.5 mL of α-amylase (75 U/mL), 25 μL de CaCl_2_ (0.3 M) and 975 μL of distilled water were used. The phase was maintained in a water bath at 37 °C, pH 7, under agitation (100 rpm) for 2 min. For the gastric phase, the volume from the oral phase was mixed with 7.5 mL of simulated gastric fluid, 1.6 mL of porcine pepsin (2000 U/mL), 5 μL of CaCl_2_ (0.3 M), 0.2 mL of HCl (1 M) and 695 μL of distilled water. The phase was maintained in a water bath at 37 °C, pH 2 under agitation (100 rpm) for 120 min. Finally, for the intestinal phase, the volume from gastric phase was mixed with 5.5mL of simulated intestinal fluid, 2.5 mL of pancreatin (100 U/mL), 1.25 mL of bile solution (10 mM), 20 μL of CaCl_2_ (0.3 M), 75 μL NaOH (1 M) and 655 μL of distilled water. The phase was maintained in a water bath at 37 °C, pH 7, under agitation (100 rpm) for 120 min. At the end of each phase (oral, gastric, and intestinal), an aliquot was collected, adjusted to pH 9 with NaOH (1 M), and placed on ice for 10 min to stop the reaction. Subsequently, the aliquot was centrifuged at 3400 rpm for 10 min, and the contents of PC, Bc, Bx and AOX (ABTS; FRAP) were analyzed.

Recovery percentage (%R) was calculated using Equation (8).

(8) $$\%R=\frac{PC,Bc,Bx\;or\;AOX\;content\;after\;oral\;or\;gastric\;phase}{Initial\;PC,Bc,Bx\;or\;AOX\;content}\times 100$$

Bioaccessibility percentage (%B) was calculated using Equation (9).

(9) $$\%B=\frac{PC,Bc,Bx\;or\;AOX\;content\;after\;intestinal\;phase}{Initial\;PC,Bc,Bx\;or\;AOX\;content}\times 100$$

### 2.8. Statistical Analysis

All analyses were performed in triplicate and statistically analyzed using Minitab 21 software by applying analysis of variance (ANOVA) and Tukey’s test to determine significant differences among means. Differences in the results with *p* ≤ 0.05 were considered statistically significant.

## 3. Results and Discussion

### 3.1. Content of Phytochemical Compounds and Antioxidant Capacity in the Concentrated Garambullo Extract

The content of phenolic compounds (Table 1) was slightly lower than that reported by Ruiz-Aguilar et al. [8]. This was probably due to the fact that the garambullo fruits used in the present study had a lower maturity stage, since Herrera-Hernández et al. [16] indicated that antioxidant capacity increases with fruit ripening.

Table 1 shows that the garambullo fruit extract exhibited a higher PC content compared with the contents of Bc and Bx, which agrees with the findings reported by Godínez-Santillán et al. [17], who mainly quantified PC in garambullo fruit extract. Likewise, the Bx content was higher (practically twice) that of Bc, which is consistent with that reported by Montiel-Sánchez et al. [2].

It should be noted that the antioxidant capacity of garambullo is mainly attributed to PC [16]; therefore, the high PC content (1399.75 ± 0.13) reported in the present study is associated with high antioxidant capacity (ABTS: 13,078.54 ± 0.22 μmol TE/100 g_dw_; FRAP: 11,917.19 ± 0.54 μmol TE/100 g_dw_).

### 3.2. Microencapsulation of the Concentrated Garambullo Extract

Concentrated garambullo extract was mixed with the wall material (gum arabic, GA) to obtain solutions containing 10%, 15% and 20% GA before drying. The yields after microencapsulation were 83.30%, 81.67% and 73.69% respectively; these results agree with those reported by Ruiz-Aguilar et al. [8]. In general, yields above 60% are considered indicative of efficient drying processes [18].

**Figure 1:**
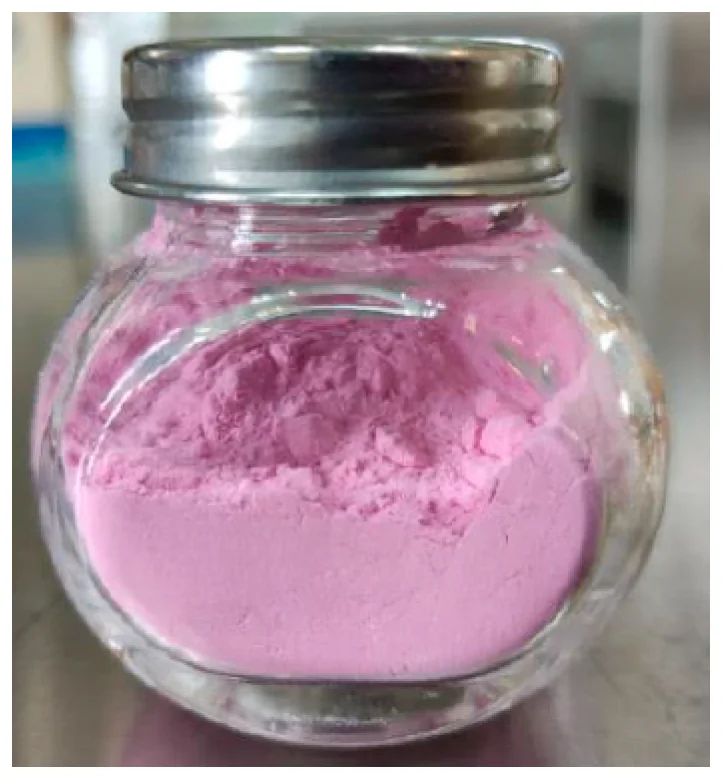
Garambullo extract microencapsulated.

Figure 1 shows the garambullo extract microencapsulated. The powders were pink in color with a soft and compact consistency.

#### 3.2.1. Retention Efficiency (%RE) and Encapsulation Efficiency (%EE)

**Figure 2:**
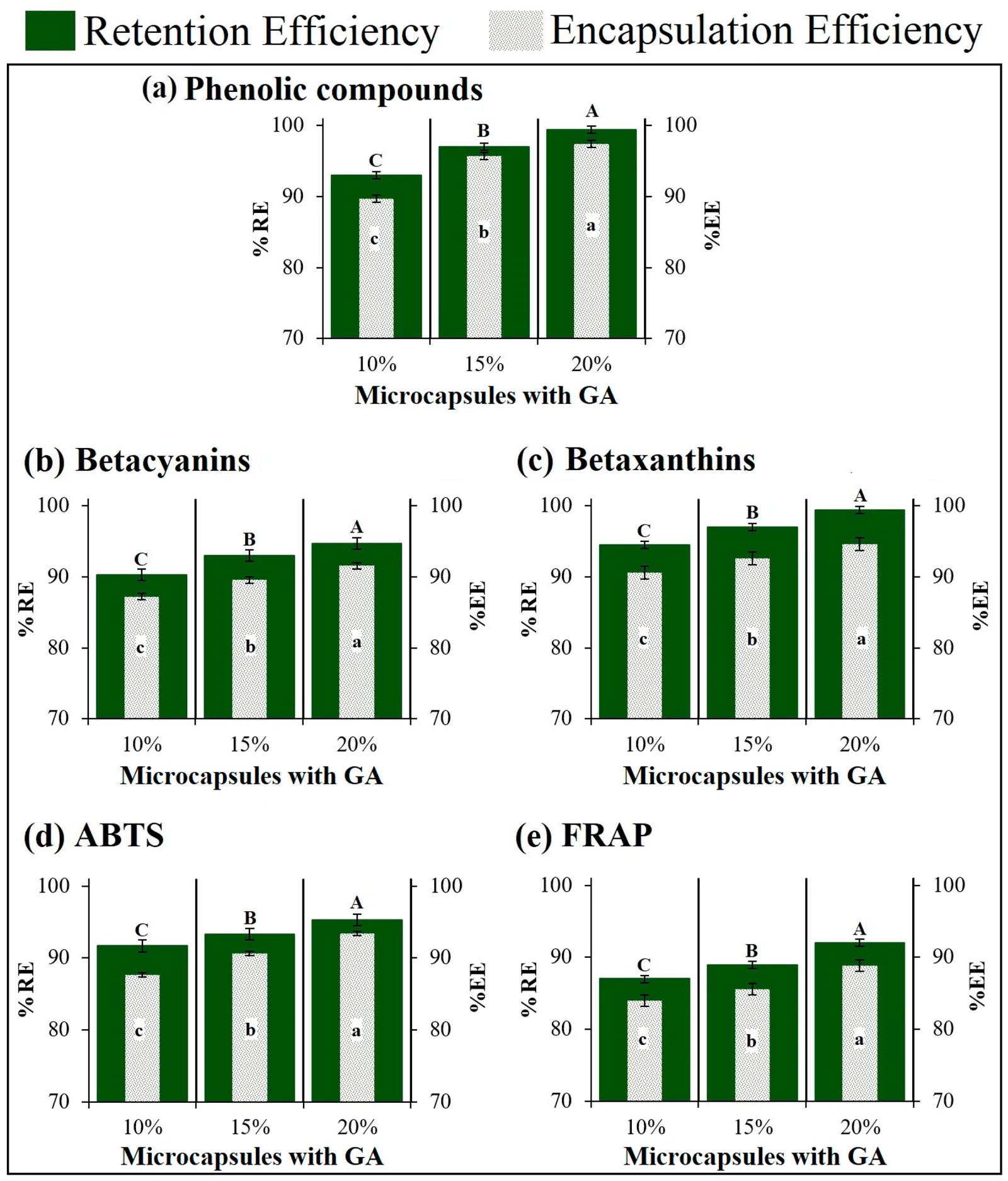
Retention efficiency (%RE) and encapsulation efficiency (%EE) of microcapsules with gum arabic (GA) at 10%, 15%, and 20%. Results are mean ± standard deviation. Means with different uppercase letters are significantly different (*p* ≤ 0.05) for RE. Means with different lowercase letters are significantly different (*p* ≤ 0.05) for EE. ABTS: 2,2′-azino-bis(3-ethylbenzothiazoline-6-sulfonic acid). FRAP: Ferric Reducing Antioxidant Power.

Figure 2 presents the RE and EE percentages of phytochemical compounds (Figure 2a–c) and antioxidant capacity (Figure 2d,e) in microcapsules with 10%, 15% and 20% GA. It can be observed that most of the retained phytochemical compounds and antioxidant capacity (≥90% for PC, Bc, Bx, and ABTS; ≥87% for FRAP) were encapsulated (≥84%).

For all phytochemical compounds and antioxidant capacity, RE and EE increased significantly (*p* ≤ 0.05) as the GA concentration increased. This trend has been reported by other authors [19], who indicated that RE and EE of the phytochemical compounds and antioxidant capacity increase as the concentration of wall material increases.

RE and EE of betaxanthins were higher than those obtained for betacyanins. This may be due to greater thermal resistance and stronger interactions between betaxanthins and GA, resulting in higher retention and encapsulation of betaxanthins during the microencapsulation process. This is consistent with Vergara-Hinostroza [20], who reported higher RE for betaxanthins than for betacyanins. Regarding RE and EE of antioxidant capacity, Figure 2 shows that FRAP exhibited lower RE and EE compared to ABTS. No reports are available on ER and EE determined by FRAP in garambullo-extract microcapsules for comparison.

#### 3.2.2. Phytochemical Compound and Antioxidant Capacity

Table 2 shows that the contents of PC, Bc, Bx and antioxidant capacity (ABTS; FRAP) of microcapsules with 10%, 15% and 20% GA decreased significantly (*p* ≤ 0.05) as the GA concentration increased (from 10% to 20%), which is due to the fact that, as the percentage of wall material increased, the amount of extract added, and therefore the content of phytochemical compounds and antioxidant capacity, is lower for the same mass of microcapsules due to a dilution effect.

#### 3.2.3. Moisture Content and Water Activity (aw)

Table 3 shows that the moisture content and aw of microcapsules with 10%, 15% and 20% GA were significantly different (*p* ≤ 0.05). Moisture content (%) of microcapsules increased as the percentage of GA increased (from 4.65% to 6.05%); by contrast, aw values of the microcapsules decreased as the wall-material concentration increased (0.31 to 0.19). This trend has been reported by other authors [21] who indicated that aw decreases as the concentration of the wall material increases. This behavior is due to the effect of GA concentration, which promotes water accumulation; however, the water available for reactions (aw) decreases because it is bound to the wall-material concentration [22].

Moisture content and aw results in microcapsules agree with those reported by other authors [8]. Also, these results are within the recommended limits (moisture content: 1–6% and aw: ≤0.5) [23], suggesting that microcapsules with 10%, 15% and 20% GA have good chemical and microbiological stability.

#### 3.2.4. Particle Size and Particle Size Distribution

Parameters determined for particle size and particle size distribution included the particle size dispersion index (SPAN) which describes the width of the particle size distribution; the Suater diameter which describes a complete distribution by relating the volume/surface area of a sphere to that of the average particle in the system; and equivalent sphere diameter, which is the diameter of a sphere with an equivalent volume.

Table 4 shows that SPAN, Suater diameter and equivalent sphere diameter of the microcapsules with 10%, 15% and 20% GA were significantly different (*p* ≤ 0.05). The SPAN, Suater diameter and equivalent sphere diameter values increased as the percentage of GA increased. This was due to the higher solids content in the solution to be dried, and also to the greater amount of dry mass, which produces larger particles.

**Figure 3:**
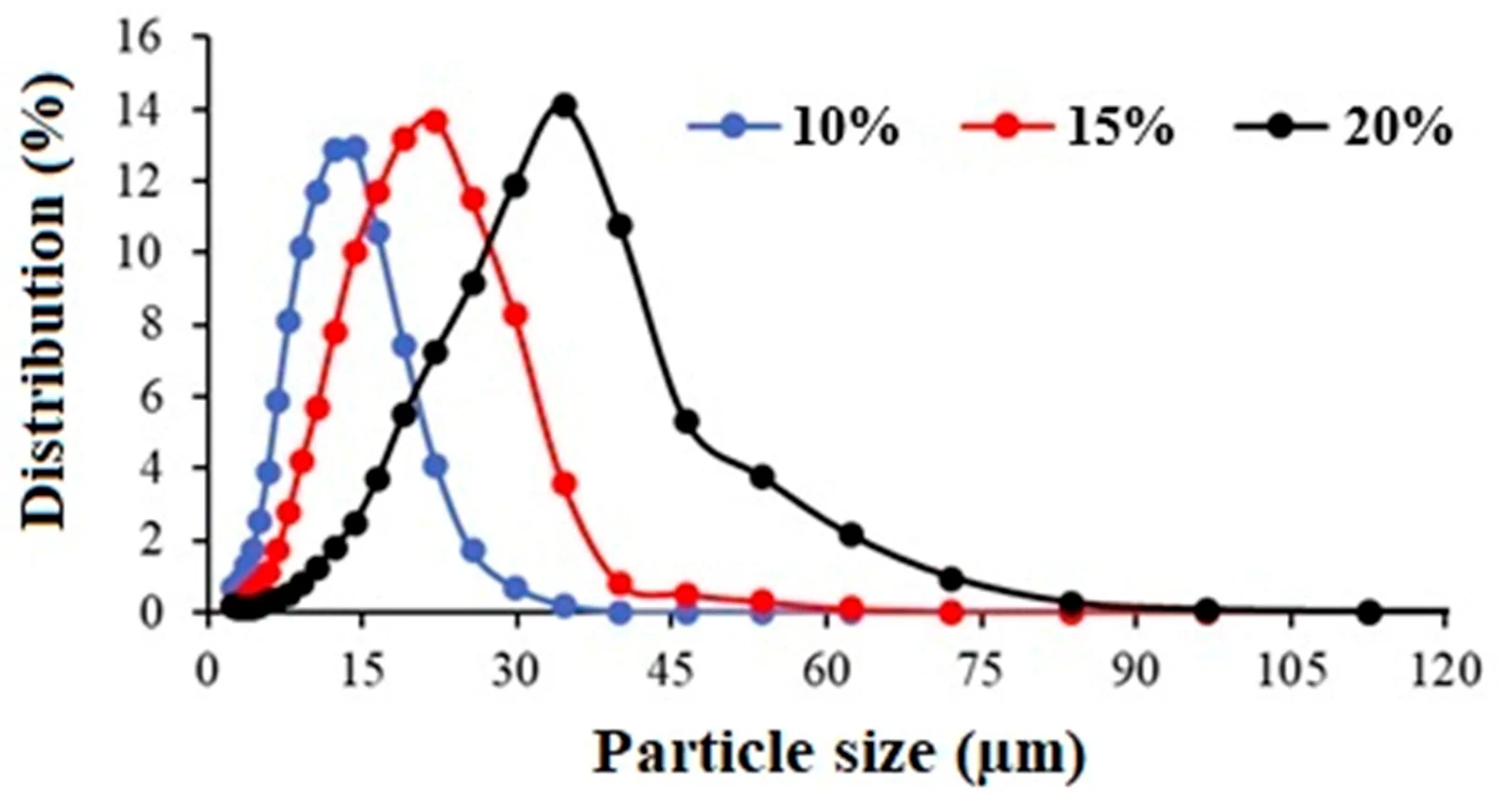
Particle size distribution of microcapsules with gum arabic (GA) at 10%, 15%, and 20%.

Figure 3 graphically shows the particle size distribution for the different concentrations of wall material used, where it can be observed that, as the concentration of wall material increased, the distribution shifted toward higher particle diameter values (µm), as did the breadth of the distribution. Operating variables in spray drying that influence particle size and particle size distribution include feed rate, outlet air temperature, feed viscosity, solids concentration, and type of atomizer, as well as the formulation of the feed fluid [24].

Particle size distribution in the microcapsules (Figure 3) is consistent with the results shown in Table 4 and with those reported by Ruiz-Aguilar et al. [8] for 10% GA used to encapsulate garambullo extract.

#### 3.2.5. Morphology

**Figure 4:**
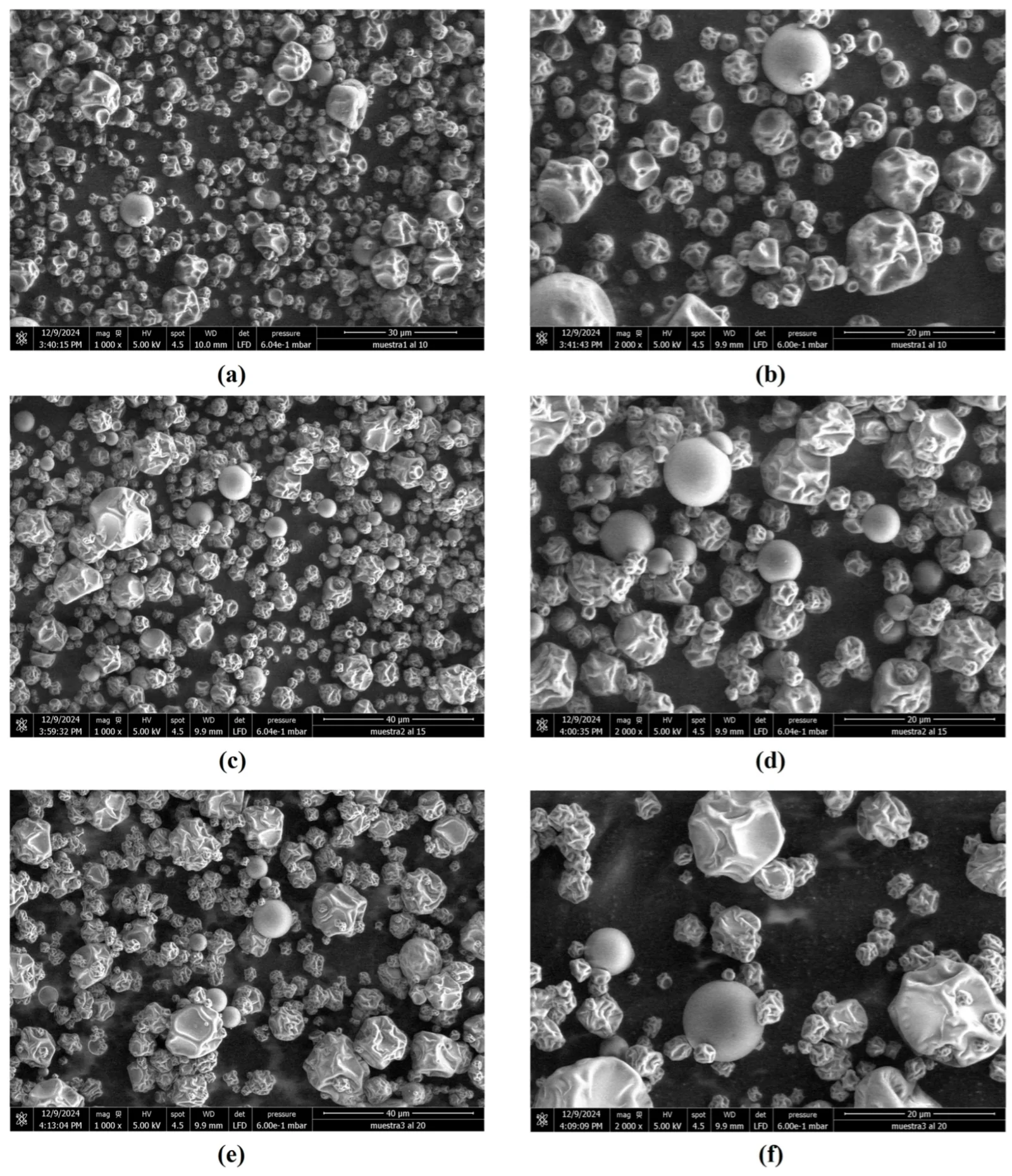
Morphology of microcapsules with gum arabic (GA) at 10%: (**a**) 1000×; (**b**) 2000×; microcapsules with GA at 15%: (**c**) 1000×; and (**d**) 2000×; and microcapsules with GA at 20%: (**e**) 1000× and (**f**) 2000×.

Figure 4a–f show that the microcapsules with 10%, 15% and 20% GA exhibited a spherical shape with wrinkled surfaces and concavities. This was due to the drying process, in which, owing to the permeability of the wall material, rapid moisture loss occurred, facilitating the transfer of water vapor out of the microparticles and causing particle shrinkage [25]. The concavities and wrinkles present in the microcapsules prevented the cracking of the wall; therefore, no particles with cracks or fractures were observed, indicating that the phytochemical compounds were adequately protected.

Figure 4b,d,f, at 2000× magnification, show that the average particle size increased as the percentage of GA increased, confirming the results described in Figure 3.

The morphology of microcapsules with different percentages of GA is similar to that reported by Ruiz-Aguilar et al. [8] for 10% GA of garambullo-extract microcapsules, who also reported shrunken spheres with wrinkled surfaces.

### 3.3. Retention of Phytochemical Compounds and Antioxidant Capacity During Storage at Different Temperature

**Figure 5:**
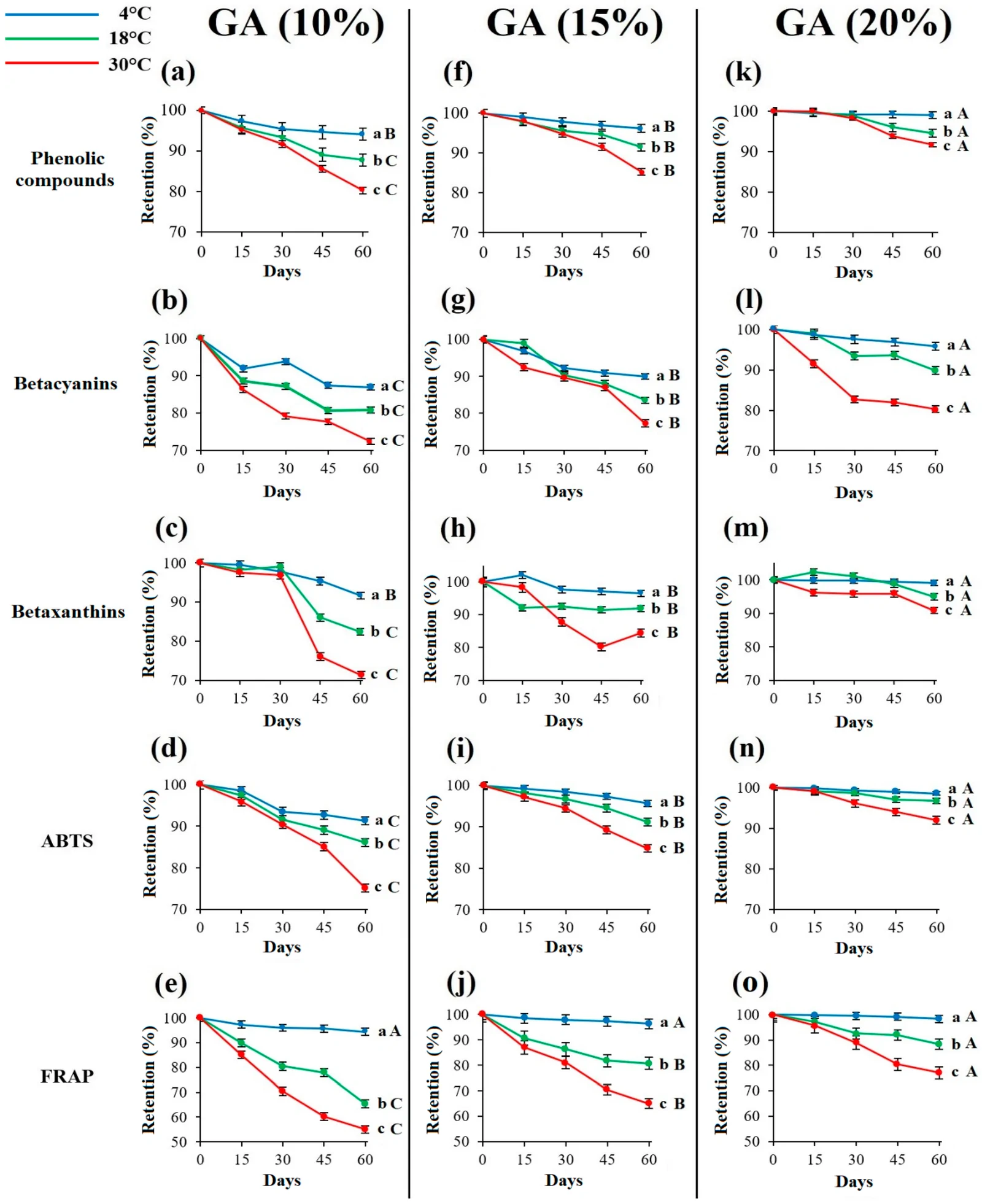
Retention (%) of phytochemical compounds and antioxidant capacity at 4 °C, 18 °C, and 30 °C of microcapsules with gum arabic (GA) at 10%: (**a**–**e**); 15%: (**f**–**j**); and 20%: (**k**–**o**). Results are mean ± standard deviation. Means with different lowercase letters are significantly different (*p* ≤ 0.05) between temperatures for the same phytochemical compound or antioxidant capacity. Means with different uppercase letters are significantly different (*p* ≤ 0.05) between GA concentrations at the same temperature. ABTS: 2,2′-azino-bis(3-ethylbenzothiazoline-6-sulfonic acid). FRAP: Ferric Reduction Antioxidant Power.

Figure 5 shows that the retention (%) of phytochemical compounds (Figure 5a–c) and antioxidant capacity (Figure 5d,e) during storage depends on both temperature (4 °C, 18 °C, and 30 °C) and wall-material concentration (10%, 15%, and 20%). For all phytochemical compounds and antioxidant capacity, at the end of the storage period (60 days), significant differences (*p* ≤ 0.05) were observed among the three storage temperatures (4 °C, 18 °C, and 30 °C) for any of the wall-material concentrations (10%, 15%, and 20%).

At the lowest temperature (4 °C) the highest retention of phytochemical compounds (≥95%) and antioxidant capacity (≥90%) was observed, regardless of wall-material concentration. However, this retention percentage decreased significantly (*p* ≤ 0.05) as the temperature increased (18 °C and 30 °C), reaching, at the end of the storage period, a loss of phytochemical compounds of up to 70% (Figure 5b,c) and a loss of antioxidant capacity of up to 55% (Figure 5e) for the 10% wall-material concentration. This retention percentage, even at the highest temperature (30 °C), was considerably higher in microcapsules with a higher wall-material concentration (Figure 5f–o).

Regarding the effect of wall-material concentration, a higher GA percentage resulted in greater retention of phytochemical compounds, with the highest retention obtained with 20% gum arabic (≥80%), regardless of storage temperature. This behavior agrees with that reported by other authors [26], who indicated that phytochemical compounds and antioxidant capacity are more stable at higher wall-material concentrations and lower storage temperatures.

With respect to degradation kinetics during storage, Table 5 presents the determination coefficients (R^2^), rate constants (*k)* and half-life times (t ½) derived from the linearization of zero-, first- and second-order reaction models obtained from the chemical kinetics of degradation of both phytochemical compounds and antioxidant capacity of microcapsules with 10%, 15% and 20% GA stored at 4 °C, 18 °C and 30 °C.

As shown in Table 5, in general, determination coefficients were ≥0.91 for the three reaction orders, indicating a good fit of the results and suggesting that any of the reaction orders analyzed could represent the degradation kinetics, since the determination coefficients were very similar to each other. Given that the zero-order model showed the lowest half-life and the second-order model the highest, the first-order model was selected as representative of the degradation of all phytochemical compounds and antioxidant capacity, as it provided an intermediate half-life value. The selected reaction order agrees with that reported by other authors [27,28,29,30] for several phytochemical compounds (phenolic compounds, anthocyanins, betalains, and carotenoids) microencapsulated with different wall-material concentrations and stored at different temperatures.

The results in Table 5 predict that, for all phytochemical compounds and antioxidant capacity, the half-life will increase as the storage temperature decreases and the GA concentration increases. This trend is consistent with that reported by Osorio et al. [27].

### 3.4. Bioaccessibility of Phytochemical Compounds and Antioxidant Capacity

**Figure 6:**
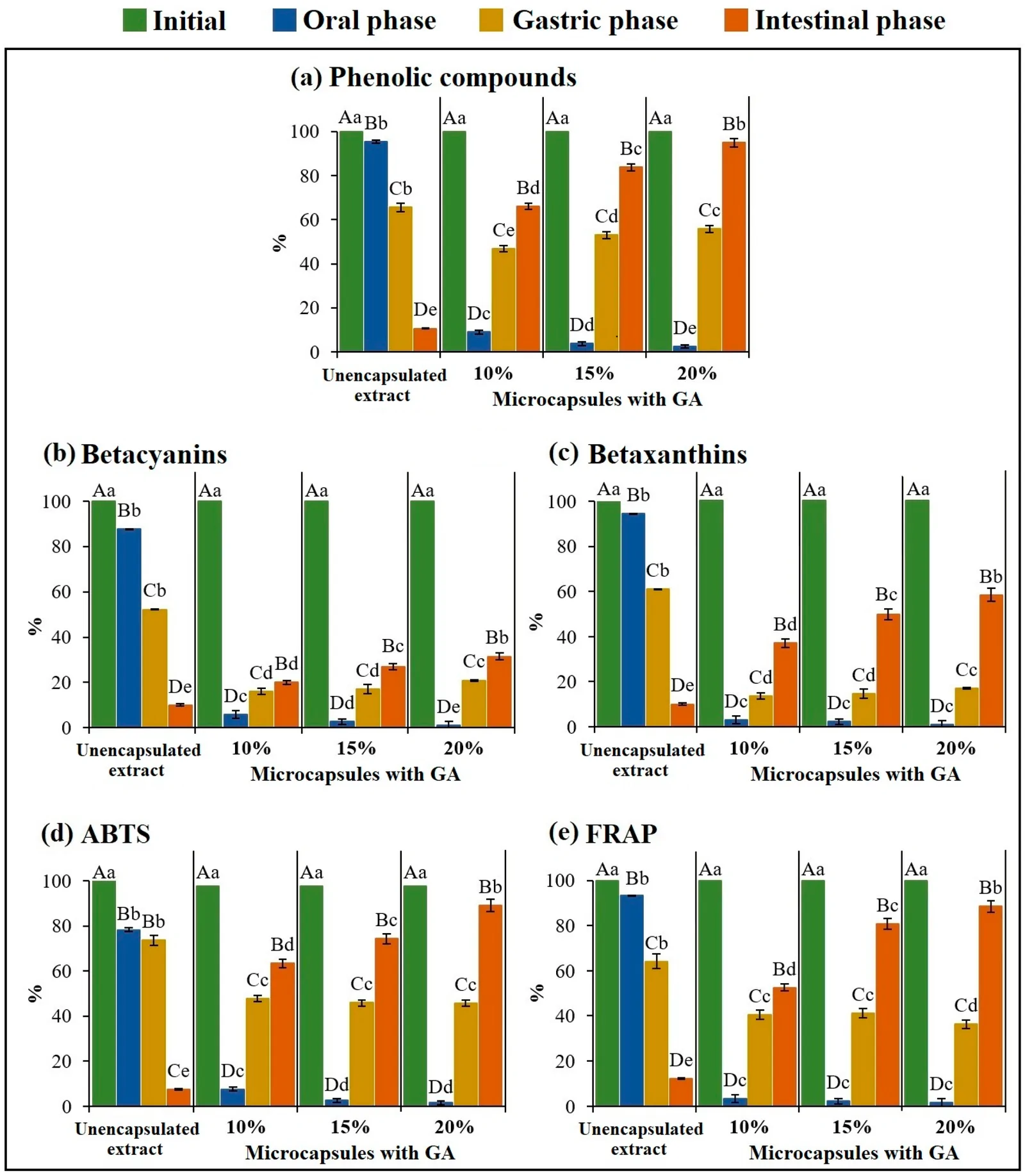
Recovery (%) and bioaccessibility (%) of unencapsulated extract and microcapsules with gum arabic (GA) at 10%, 15%, and 20%. Results are mean ± standard deviation. Means with different capital letters are significantly different (*p* ≤ 0.05) in the same sample. Means with different lowercase letters are significantly different (*p* ≤ 0.05) in the same phase (oral, gastric, and intestinal). ABTS: 2,2′-azino-bis(3-ethylbenzothiazoline-6-sulfonic acid). FRAP: Ferric Reduction Antioxidant Power.

The recovery and bioaccessibility percentages of phytochemical compounds and antioxidant capacity of non-encapsulated extract, used as reference, and of microcapsules with different wall-material concentrations, are presented in Figure 6a–e. Initial content was considered as 100%. For all phytochemical compounds and antioxidant capacity, there was a significant difference (*p* ≤ 0.05) in each phase (oral, gastric, and intestinal) for the same sample (non-encapsulated extract or microcapsules); likewise, there was a significant difference (*p* ≤ 0.05) when considering the same phase among the different samples.

For the non-encapsulated extract, it can be observed that, for all phytochemical compounds (Figure 6a–c) and antioxidant capacity (Figure 6d,e), recovery percentage (oral and gastric phases) and bioaccessibility percentage (intestinal phase) decreased significantly (*p* ≤ 0.05) relative to the initial content as digestion progressed from the oral to the gastric phase. This was because the phytochemical compounds were not protected as they were in the microcapsules and, therefore, degraded more rapidly and easily, which was reflected in their drastic decrease and in the reduction in antioxidant capacity (Figure 6a–e).

In the oral phase, phytochemical compounds interact with the enzyme α-amylase, which favors their release and rapid degradation [31]. Likewise, the hydrophilic nature of certain compounds, such as betalains, promotes their degradation despite the short duration (2 min) of the oral phase. In the gastric phase, 40–60% of phytochemical compounds are generally degraded due to the hydrolysis of compounds bound to proteins or carbohydrates, in addition to the change from alkaline pH (pH 7 in the oral phase) to acidic pH (pH 2 in the gastric phase), as well as enzymatic action, which favors the transformation or degradation of phytochemical compounds [32,33]. Finally, in the intestinal phase, the low bioaccessibility of phytochemical compounds (PC: 10.63%; Bc: 10.04%; and Bx: 10.04%) of non-encapsulated extract may be attributed to the transition from the acidic gastric environment to the neutral intestinal environment, incubation time (2 h in the gastric phase and 2 h in the intestinal phase, 4 h in total), incubation temperature (37 °C), and the loss, degradation or transformation of phytochemical compounds due to the action of bile salts and enzymes [34]; this leads to a decrease in bioaccessible phytochemical compounds [35] and, consequently, to low bioaccessibility of antioxidant capacity (ABTS: 7.52%; FRAP: 12.19%).

The results described above agree with those reported by other authors [8,36,37], who indicated that non-microencapsulated fruit extracts generally exhibit considerable losses (≥90%) in the bioaccessibility of phytochemical compounds after in vitro digestion; therefore, protecting these compounds is of great importance.

With respect to the microcapsules (10%, 15% and 20% GA), Figure 6 shows that, for all phytochemical compounds (Figure 6a–c) and antioxidant capacity (Figure 6d,e), recovery percentage (oral and gastric phases) and bioaccessibility percentage (intestinal phase) increased significantly (*p* ≤ 0.05) compared with the non-encapsulated extract. This behavior was due to the fact that the wall material protected the phytochemical compounds, which were gradually released during the in vitro digestion process.

As shown in Figure 6a–e, in the microcapsules, the release of phytochemical compounds and retention of antioxidant capacity were associated with GA concentration (10%, 15% and 20%); the higher the GA percentage, the greater the protection of phytochemical compounds and antioxidant capacity obtained during digestion.

In the oral phase, the recovery percentage of phytochemical compounds for the three GA concentrations was very low (≤10%), since the wall material acted as a barrier that protected the phytochemical compounds, preventing their release and degradation. In the gastric phase, the recovery percentage of phenolic compounds and betalains (betacyanins and betaxanthins) increased gradually and significantly (*p* ≤ 0.05) compared with the oral phase, because the compounds were partially and controllably released due to the wall material. In general, the gastric environment (pH 2), enzymatic action (pepsin), and temperature may favor the transformation or degradation of phytochemical compounds [15]. For example, betalains (betacyanins and betaxanthins) are stable at pH 5–6; however, at high temperature and extreme pH values (both acidic and basic) decarboxylation, molecular cleavage, protonation, formation of other products, and degradation occur. Therefore, the use of gum arabic served to protect phenolic compounds and betalains during the gastric phase [38,39].

Roman-Maldonado et al. [40] indicated that polysaccharides (pectins, mucilages, and gums) stabilize the structure of betalains; this confirms that microencapsulation with gum arabic increased the complexity of the matrix, protecting these phytochemical compounds and preventing their degradation.

Microencapsulation of antioxidant compounds using GA was effective because, being composed of complex polysaccharides, GA is not hydrolyzed by the pepsin enzyme used during the gastric phase. As a result, the phytochemical compounds are protected during this stage of the digestive process for their subsequent release in the intestinal phase [41]. Tonon et al. [42] indicated that phytochemical compounds encapsulated with gum arabic can be classified as “slow release” compounds, since this type of wall material protects encapsulated compounds during the gastric phase.

Other important factors that contributed to the protection of phytochemical compounds using GA are related to its physicochemical and rheological properties. During the gastric phase, GA is deionized, and negatively charged pepsin generates electrostatic repulsion with GA, which represents a steric hindrance and prevents the release of phytochemical compounds [9]. In addition, the structural configuration and mechanical resistance of GA are not altered when the pH changes from 7 (oral phase) to 2 (gastric phase) [43].

Finally, in the intestinal phase, the microcapsules exhibited good bioaccessibility of phenolic compounds (PC: ≥70%) due to the protective effect of GA in the microencapsulated extracts, which acted as a physical barrier, effectively protecting and releasing phytochemical compounds in the intestinal phase. This was reflected in the good bioaccessibility of antioxidant capacity (ABTS: 65%; FRAP: 55%). Agredano-De la Garza et al. [32] indicated that the increase in antioxidant capacity at the end of the intestinal phase is due to the pH change from the gastric phase (pH 2) to the intestinal phase (pH 7), which modifies the ionization of the hydroxyl groups of phenolic compounds, improving their ability to donate electrons or hydrogen and thereby increasing antioxidant capacity. This agrees with Ordoñez-Díaz et al. [44] who indicated that phytochemical release is due to the fact that pH and pancreatin activity break non-covalent bonds (hydrogen bonds, Van der Waals forces, and hydrophobic or ionic interactions) between the food matrix and low-molecular-weight phytochemical compounds, releasing them and consequently, increasing bioaccessibility.

Protection and release of PC, Bc, Bx and AOX were significantly different (*p* ≤ 0.05) in each phase of in vitro digestion, and the release increased with GA concentration. Therefore, the concentration of this wall material improved the protection and release of phytochemical compounds during the different stages of the simulated gastrointestinal process.

It is important to mention that different factors influence the effective release and stability of encapsulated compounds. For example, good protection by the wall material during digestion is related to high encapsulation efficiency [41]. In this regard, the highest encapsulation efficiencies were obtained in microcapsules with 20% GA (Figure 2).

Finally, the bioaccessibility of antioxidant capacity determined by ABTS (89.17%) was very similar to that obtained by FRAP (88.46%), although %RE and %EE values were lower for FRAP (Figure 2e) than for ABTS (Figure 2d).

In summary, the results presented in Figure 6a–e indicate that the phytochemical compounds and antioxidant capacity of garambullo extract were protected by microencapsulation using gum arabic and that the effect of increasing the wall material significantly improved the bioaccessibility of the compounds and, consequently, antioxidant capacity.

## 4. Conclusions

This study demonstrated that garambullo extract microencapsulated with the highest wall-material concentration (20%) exhibited the highest encapsulation efficiency and protection of phytochemical compounds and antioxidant capacity. During storage, microcapsules with the highest wall-material concentration (20%) and stored at the lowest temperature (4 °C) showed the greatest stability and half-life of phytochemical compounds and antioxidant capacity. Likewise, after the simulated gastrointestinal process, the bioaccessibility of phytochemical compounds and antioxidant capacity was higher in the microcapsules with the highest wall-material concentration (20%). Depending on the potential use of the microcapsules, it is important to consider the retention percentage, wall-material concentration, and bioaccessibility of phytochemical compounds and antioxidant capacity. Thus, for the development of functional foods with a relatively short shelf life, the use of 10% GA is recommended because of the higher concentration of phytochemical compounds per gram of capsules; however, for nutraceutical products with a longer shelf life and stored at room temperature, the use of 20% GA is recommended because of the longer half-life achieved during storage.

## Figures and Tables

**Table 1:** Phytochemical content and antioxidant capacity of concentrated garambullo extract.

Parameter	Content
PC (mg GAE/100 g_dw_)	1399.75 ± 5.13
Bc (mg/100 g_dw_)	29.03 ± 0.23
Bx (mg/100 g_dw_)	60.54 ± 0.10
ABTS (μmol TE/100 g_dw_)	13,078.54 ± 8.22 ^a^
FRAP (μmol TE/100 g_dw_)	11,917.19 ± 6.54 ^b^

Results are the mean ± standard deviation. Means with different letters are significantly different (*p* ≤ 0.05) in antioxidant capacity. PC: phenolic compounds. Bc: betacyanins. Bx: betaxanthins. ABTS: 2,2′-azino-bis(3-ethylbenzothiazoline-6-sulfonic acid). FRAP: Ferric Reduction Antioxidant Power.

**Table 2:** Phytochemical content and antioxidant capacity of microcapsules with gum arabic (GA) at 10%, 15% and 20%.

Parameter	Microcapsules		
	GA at 10%	GA at 15%	GA at 20%
PC (mg GAE/100 g_dw_)	665.29 ± 2.68 ^a^	445.33 ± 5.97 ^b^	281.15 ± 3.44 ^c^
Bc (mg/100 g_dw_)	4.06 ± 0.58 ^a^	2.89 ± 0.37 ^b^	1.23 ± 0.06 ^c^
Bx (mg/100 g_dw_)	5.22 ± 0.84 ^a^	3.17 ± 0.69 ^b^	1.42 ± 0.12 ^c^
ABTS (μmol TE/100 g_dw_)	5686.29 ± 6.18 ^a,A^	4696.76 ± 5.27 ^b,A^	1915.38 ± 2.49 ^c,B^
FRAP (μmol TE/100 g_dw_)	2849.93 ± 5.34 ^a,B^	2210.56 ± 6.14 ^b,B^	1995.93 ± 5.36 ^c,A^

Results are the mean ± standard deviation. Means with different lowercase letters per row are significantly different (*p* ≤ 0.05). Means with different uppercase letters per column are significantly different (*p* ≤ 0.05) in antioxidant capacity. PC: phenolic compounds. Bc: betacyanins. Bx: betaxanthins. ABTS: 2,2′-azino-bis(3-ethylbenzothiazoline-6-sulfonic acid). FRAP: Ferric Reduction Antioxidant Power.

**Table 3:** Moisture and water activity of microcapsules with gum arabic (GA) at 10%, 15% and 20%.

Microcapsules	Moisture (%)	Water Activity
GA at 10%	4.65 ± 0.01 ^c^	0.31 ± 0.00 ^a^
GA at 15%	5.08 ± 0.09 ^b^	0.26 ± 0.00 ^b^
GA at 20%	6.05 ± 0.07 ^a^	0.19 ± 0.00 ^c^

Results are the mean ± standard deviation. Means with different letters per column are significantly different (*p* ≤ 0.05).

**Table 4:** Particle size of microcapsules with gum arabic (GA) at 10%, 15% and 20%.

Microcapsules	SPAN	Sauter Diameter (D_[3,2]_) µm	Equivalent Sphere Diameter (D_[4,3]_) µm
GA at 10%	1.11 ± 0.04 ^c^	8.22 ± 0.06 ^c^	11.64 ± 0.07 ^c^
GA at 15%	1.21 ± 0.06 ^b^	13.79 ± 0.03 ^b^	18.57 ± 0.04 ^b^
GA at 20%	1.35 ± 0.02 ^a^	19.26 ± 0.08 ^a^	27.01 ± 0.07 ^a^

Results are the mean ± standard deviation. Means with different letters per column are significantly different (*p* ≤ 0.05).

**Table 5:** Rate constants (*k*) and half-life (t ½) for reaction orders 0, 1 and 2 for the degradation of phytochemical compounds and antioxidant capacity of microcapsules with gum arabic (GA) at 10%, 15% and 20% stored at 4 °C, 18 °C and 30 °C.

			Microcapsules								
			GA at 10%			GA at 15%			GA at 20%		
Parameter	Temperature	Reaction Orden	R^2^	*k* (kmol K)	t ½ (Days)	R^2^	*k* (kmol K)	t ½ (Days)	R^2^	*k* (kmol K)	t ½ (Days)
PC	4 °C	0	0.91	0.64	515.73	0.99	0.29	760.13	0.91	0.05	277.82
		1	0.91	0.31	693.15	0.99	0.47	990.21	0.91	0.20	3465.72
		2	0.91	0.40	751.55	0.99	0.85	1122.16	0.92	0.38	5916.25
	18 °C	0	0.97	1.56	240.98	0.98	0.63	368.98	0.91	0.36	396.76
		1	0.97	0.67	315.07	0.98	0.93	495.11	0.91	0.91	533.19
		2	0.98	0.49	440.88	0.97	0.56	711.78	0.92	0.79	735.02
	30 °C	0	0.99	2.18	152.37	0.95	1.07	207.71	0.90	0.32	387.92
		1	0.98	0.28	187.34	0.94	0.02	266.65	0.90	0.98	533.19
		2	0.98	0.79	250.52	0.93	0.46	374.05	0.90	0.82	662.01
Bc	4 °C	0	0.91	0.05	481.18	0.97	0.93	788.04	0.91	0.34	834.31
		1	0.96	0.01	683.34	0.94	0.02	1002.91	0.91	0.78	1179.38
		2	0.93	0.03	987.34	0.92	0.85	1932.05	0.91	0.92	2034.82
	18 °C	0	0.93	0.02	359.51	0.91	0.73	310.04	0.96	0.74	598.25
		1	0.98	0.09	458.36	0.91	0.65	567.06	0.96	0.52	653.21
		2	0.94	0.05	568.21	0.92	0.47	761.50	0.97	0.32	986.10
	30 °C	0	0.97	0.04	107.35	0.92	0.02	198.02	0.90	0.84	298.14
		1	0.98	0.07	258.61	0.92	0.56	358.19	0.90	0.67	334.58
		2	0.97	0.09	384.23	0.91	0.65	583.24	0.90	0.96	358.81
Bx	4 °C	0	0.93	0.06	590.91	0.91	0.92	798.94	0.91	0.80	1135.29
		1	0.93	0.07	770.16	0.92	0.65	1020.35	0.91	0.72	1732.87
		2	0.92	0.04	968.38	0.92	0.91	1438.53	0.91	0.46	2590.67
	18 °C	0	0.91	0.02	270.65	0.91	0.34	420.14	0.96	0.61	557.89
		1	0.92	0.07	346.57	0.91	0.65	770.16	0.96	0.53	770.16
		2	0.91	0.03	459.56	0.91	0.18	826.45	0.97	0.72	1179.25
	30 °C	0	0.99	0.04	24.02	0.92	0.02	290.02	0.91	0.96	320.63
		1	0.99	0.07	155.61	0.99	0.08	495.11	0.91	0.83	433.22
		2	0.99	0.01	192.54	0.90	0.34	795.83	0.91	0.46	618.81
ABTS	4 °C	0	0.93	0.05	390.46	0.91	0.52	598.81	0.93	0.27	2353.66
		1	0.94	0.04	533.19	0.91	0.64	415.83	0.93	0.76	2590.67
		2	0.94	0.03	760.86	0.91	0.94	504.21	0.93	0.74	3465.74
	18 °C	0	0.96	0.09	216.18	0.93	0.92	403.60	0.96	0.57	979.40
		1	0.96	0.03	277.26	0.93	0.63	770.16	0.96	0.15	1037.79
		2	0.97	0.04	358.76	0.93	0.80	693.15	0.96	0.83	1386.29
	30 °C	0	0.91	0.06	331.34	0.96	0.03	366.70	0.95	0.04	994.85
		1	0.92	0.06	433.22	0.97	0.60	408.69	0.95	0.33	1386.29
		2	0.93	0.08	572.48	0.97	0.94	693.15	0.95	0.25	1635.58
FRAP	4 °C	0	0.91	0.05	324.41	0.96	0.36	621.12	0.91	0.11	1365.56
		1	0.91	0.07	584.91	0.96	0.97	871.21	0.91	0.19	1821.64
		2	0.91	0.09	770.16	0.96	0.62	1155.25	0.91	0.82	2310.49
	18 °C	0	0.97	0.35	38.50	0.91	0.11	85.78	0.96	0.19	261.76
		1	0.96	0.57	92.36	0.93	0.67	171.19	0.96	0.42	267.52
		2	0.95	0.37	105.02	0.90	0.29	210.04	0.96	0.38	346.57
	30 °C	0	0.98	0.55	21.01	0.98	0.34	26.66	0.98	0.12	63.78
		1	0.99	0.01	61.58	0.96	0.91	68.53	0.97	0.00	107.18
		2	0.99	0.73	62.45	0.94	0.52	72.2	0.96	0.00	128.36

PC: Phenolic compounds. Bc: Betacyanins. Bx: Betaxanthins. ABTS: 2,2′-azino-bis(3-ethylbenzothiazoline-6-sulfonic acid). FRAP: Ferric Reduction Antioxidant Power.

## Data Availability

The original contributions presented in the study are included in the article; further inquiries can be directed to the corresponding authors.
